# Exploring multidimensional determinants of medication error reporting in China: a qualitative study using the theoretical domains framework

**DOI:** 10.3389/fphar.2025.1590794

**Published:** 2025-06-10

**Authors:** Hui Guo, Qian Guo, Xiuping Wang, Yonghong Liu, Ze Wang

**Affiliations:** ^1^ Shanxi Cardiovascular Hospital, Department of Pharmacy, Taiyuan, Shanxi, China; ^**2**^ Second Hospital of Shanxi Medical University, Department of Pharmacy, Taiyuan, Shanxi, China

**Keywords:** medication error, error reporting, qualitative, theoretical domains framework, China

## Abstract

**Objective:**

Medication errors, defined as preventable events leading to inappropriate medication use or patient harm, remain a critical global healthcare challenge. Despite regulatory mandates in China, underreporting remains systemic. Existing research on healthcare professionals’ reporting behaviors has been constrained by reliance on isolated theoretical frameworks, which inadequately address the interplay of individual, organizational, and cultural determinants. The aim of this study is to systematically explore barriers and facilitators affecting medication error reporting among Chinese healthcare professionals, using the Theoretical Domains Framework (TDF), a comprehensive behavioral model synthesizing 14 domains.

**Methods:**

We conducted semi-structured interviews with 27 nurses, pharmacists, and physicians in Chinese hospitals (July-October 2024). Interviews were guided by the TDF to explore barriers to medication error reporting. Data were analyzed through framework analysis using NVivo 14.0 until thematic saturation was reached.

**Results:**

Analysis of semi-structured interviews explored 14 Theoretical Domains Framework (TDF) domains influencing medication error reporting among Chinese healthcare professionals, with 21 barriers and 15 facilitators delineated. Notably, eight domains emerged as predominant barriers: Knowledge, Skills, Beliefs about capabilities, Beliefs about consequences, Memory, attention and decision processes, Environmental context and resources, Social influences, and Emotion.

**Conclusion:**

This study highlights the interplay of individual, cultural, and systemic factors shaping medication error reporting in China. The TDF effectively identified context-specific barriers, including knowledge fragmentation, fear of career repercussions, and inefficient reporting systems. To foster a safety culture, interventions must integrate targeted training, simplified reporting mechanisms, and leadership commitment to non-punitive environments. Cross-professional collaboration and policy enforcement are critical to transforming reporting from a fragmented obligation into a sustainable safety practice. These insights offer a roadmap for optimizing error reporting systems in hierarchical healthcare settings globally.

## 1 Introduction

Medication errors, defined as “any preventable event that may cause or lead to inappropriate medication use or patient harm” by the US National Coordinating Council for Medication Error Reporting and Prevention (NCCMERP), represent a critical global public health challenge with profound economic and societal consequences. According to the World Health Organization (WHO), approximately 1 in 10 patients worldwide experiences harm during medical care, with over 3 million annual deaths attributed to unsafe practices (Slawomirski, 2020). In low- and middle-income countries, unsafe medical care accounts for 4% of total mortality, reflecting systemic vulnerabilities in healthcare systems (Slawomirski, 2020). Alarmingly, over 50% of medical harm is preventable, with nearly half linked to medication-related errors (Panagioti et al., 2019). Globally, medication errors alone incur direct economic losses of $42 billion annually, alongside indirect healthcare costs of $140 billion, affecting more than 7 million patients (Tariq et al., 2025). These staggering figures underscore the urgent need to prioritize medication safety as a cornerstone of pharmaceutical management.

Recognizing this imperative, governments worldwide have implemented measures to mitigate medication errors. Countries like the United States, the United Kingdom, and Canada have established robust reporting systems supported by standardized tools for error monitoring, evaluation, and prevention (Network, 2023). In China, regulatory efforts include the Regulations on Pharmaceutical Affairs Management in Medical Institutions (2011) mandated error reporting systems, while subsequent policies-such as the Implementation Details of the Standards for Tertiary General Hospitals (2012) and the Quality Control Indicators for Pharmaceutical Management (2020)-designated medication error reporting rates as a key performance metric (China, 2014). Despite these initiatives, underreporting persists as a systemic issue. For instance, in 2023, only 439 healthcare facilities across 26 Chinese provinces reported 27,742 medication errors, highlighting widespread noncompliance and critical gaps in implementation (Zhang et al., 2024).

Existing research on healthcare professionals’ medication error reporting behavior remains fragmented. While prior studies have identified barriers such as fear of punishment (Chiang et al., 2010), insufficient training (Sarvadikar et al., 2010), knowledge gaps (Alqubaisi et al., 2016), and unsupportive organizational cultures (Stewart et al., 2018), their reliance on isolated theoretical frameworks—such as the Theory of Planned Behavior (TPB) (Natan et al., 2017) or the Knowledge-Attitude-Practice (KAP) model (Yousef et al., 2021)—limits holistic insights. These models often exhibit conceptual overlap (e.g., conflating “behavioral attitudes” and “attitudes”) and fail to account for the multifactorial interplay of personal, interpersonal, and environmental influences on reporting behavior. To address these limitations, this study adopts the Theoretical Domains Framework (TDF), a comprehensive framework synthesizing 33 behavioral theories into these 14 domains: knowledge, skills, social/professional role and identity, beliefs about capabilities, optimism, beliefs about consequences, reinforcement, intentions, goals, memory, attention and decision processes, environmental context and resources, social influences, emotion, and behavioral regulation (Michie et al., 2005; Cane et al., 2012).

The TDF has proven effective in healthcare research for identifying barriers to adopting new systems. For example, Stewart et al. identified fear of repercussions, concerns about confidentiality, and potential impact on career progression as key barriers to medication error reporting in Qatar (Stewart et al., 2018). Similarly, Alqubaisi et al. highlighted lack of feedback and time constraints as critical obstacles in the United Arab Emirates’ context (Alqubaisi et al., 2016). However, China’s unique sociocultural context necessitates tailored investigations. Traditional values emphasizing hierarchical structures, collectivism, and respect for authority profoundly shape hospital dynamics (Hu et al., 2024), often discouraging open communication and error reporting (Hodkinson et al., 2022). Compounding this issue, China’s healthcare environment faces escalating doctor-patient tensions, with 62% of physicians encountering medical disputes and 18,670 healthcare liability cases reported in 2020 alone—a 3% annual increase (Gao et al., 2024). These cultural and systemic pressures create a complex landscape where medication error reporting is further stifled by fear of litigation and reputational damage.

This study aims to address these gaps by applying the TDF to systematically explore behavioral factors influencing medication error reporting among Chinese healthcare professionals. By examining their perspectives through this integrative lens, the study seeks to generate actionable insights for optimizing reporting systems and fostering a culture of safety in China’s evolving healthcare environment.

## 2 Methods

This qualitative study employed face-to-face, semi-structured interviews to explore healthcare professionals’ experiences and perspectives on medication error reporting in China. The design prioritized capturing rich, contextual insights into the barriers and facilitators of reporting behaviors within real-world clinical settings.

### 2.1 Setting

The study was conducted in 3 secondary and 3 tertiary hospitals across Shanxi Province, China. These institutions operate under national medication safety regulations, including the Regulations on Pharmaceutical Affairs Management in Medical Institutions (2011) and the Quality Control Indicators for Pharmaceutical Management (2020), which mandate reporting of all medication error-including those intercepted before reaching patients.

### 2.2 Recruitment and sampling

Purposive sampling was utilized to select interviewees who had shown interest in the study via a preliminary questionnaire. This approach ensured representation across various healthcare professions, years of experience, and professional titles. Interviewees were individually contacted by telephone to arrange the interview’s date, time, and venue.

### 2.3 Sample size

Sampling continued until thematic saturation was achieved, following the guidelines by Francis et al. (2010). Initial interviews included five interviewees per profession (nurses, pharmacists, physicians). Recruitment ceased when no new themes emerged over three consecutive interviews within each professional category. The final sample consisted of 27 interviewees: 9 nurses, 10 pharmacists, and 8 physicians, representing diverse demographic and professional backgrounds (detailed in Table 1).

**TABLE 1:** Sociodemographic characteristics of study participants.

Characteristic	Mean ± SD or n (%)
Sex	
Male	6 (22.2)
Female	21 (77.8)
Age (years)	371 ± 8.5
Years in service	12.1 ± 8.8
Education Level	
Junior College	2 (7.4)
Bachelor’s	11 (40.7)
Master’s	11 (40.7)
Doctorate	3 (11.1)
Hospital Level	
Secondary Class A	9 (33.3)
Tertiary Class B	11 (40.7)
Tertiary Class A	7 (25.9)
Professional Title	
Junior	8 (29.6)
Intermediate	9 (33.3)
Senior	10 (37.0)

### 2.4 Data generation

The semi-structured interview guide was developed using the Theoretical Domains Framework (TDF), with iterative refinements informed by three patient safety experts and two researchers with expertise in qualitative methods (Supplementary Table S1). To ensure practical applicability, the guide underwent three profession-specific pilot interviews (nurse, pharmacist, physician), followed by adjustments to question clarity and clinical relevance. Interviews were conducted in Mandarin by researcher GQ (as part of her doctoral studies) with specialized training in phenomenological interviewing methodologies and 15 years of extensive clinical pharmaceutical practice at a tertiary care hospital in China. Data were collected between July and October 2024, with all interviews audio-recorded and transcribed in full using a naturalistic approach to capture every utterance in precise detail. All interviewees were afforded the opportunity to review their transcripts prior to analysis. GH and GQ reviewed the first five audio-recordings to ensure high-quality interviewing skills and thus promote data credibility and checked the reliability of transcribing of each interview. Furthermore, a very clear audit trail was maintained with documented details of data gathering to promote dependability.

### 2.5 Data analysis

All interviews were verbatim transcribed within 48 h using Microsoft Word 2023, after which two researchers (GH and GQ) independently cross-checked transcripts against audio recordings and consulted participants for clarification as needed. The verified transcripts were analyzed in NVivo 14.0 (QSR International) using Braun and Clarke’s thematic framework (Braun and Clarke, 2006), guided by the TDF. framework approach, guided by the TDF. The analysis involved: (1) immersive transcript review to achieve saturation; (2) extraction of 313 medication error reporting-relevant statements; (3) inductive synthesis into 31 themes; (4) TDF-aligned thematic clustering; (5) expert (WXP)-guided refinement with operational definitions; (6) iterative consolidation via constant comparison; and (7) multi-modal validation (telephone/WeChat/email) to confirm theme representativeness. GH and GQ conducted independent analyses with phased cross-verification; discrepancies were resolved by a medication safety specialist (WZ, 33 years’ experience). Final themes underwent plenary review to ensure coherence and fidelity.

### 2.6 Ethical considerations

Ethical approval was obtained from the Ethics Committee of the Second Hospital of Shanxi Medical University (Ref no: 2023-YX-202). All interviewees provided informed consent, and confidentiality was strictly maintained throughout the research process.

## 3 Results

A total of 27 healthcare professionals participated in this study. The cohort comprised 21 females (77.8%) and 6 males (22.2%), with ages ranging from 25 to 54 years (37.07 ± 8.49 years). The sample included professionals from diverse hospital tiers: 9 (33.3%) from secondary Class A hospitals, 11 (40.7%) from tertiary Class B hospitals, and 7 (25.9%) from tertiary Class A hospitals. Educational backgrounds varied, with 2 interviewees (7.4%) holding associate degrees, 11 (40.7%) bachelor’s degrees, 11 (40.7%) master’s degrees, and 3 (11.1%) doctorates. Professional titles were distributed as follows: 8 (29.6%) junior titles, 9 (33.3%) intermediate titles, and 10 (37.0%) senior titles. Full sociodemographic details are provided in Table 1.

Through in-depth interviews and thematic analysis, 14 themes emerged regarding factors influencing medication error reporting. These themes, organized within the Theoretical Domains Framework (TDF), encompassed both facilitators and barriers to reporting behaviors.Barrier-related factors comprised 21 subthemes across the domains of knowledge, skills, beliefs in capabilities, optimism, beliefs about consequences, intentions, goals, memory/attention and decision processes, environmental context and resources, social influences, and emotions. Facilitator-related factors included 15 subthemes within the domains of knowledge, skills, social/professional role and identity, beliefs in capabilities, optimism, beliefs about consequences, reinforcement, intentions, goals, environmental context and resources, and behavioral regulation. A comprehensive presentation of themes, sub-themes, and their alignment with TDF domains is provided in Table 2.

**TABLE 2:** Themes and sub-themes of factors influencing medication error reporting among healthcare professionals.

Theoretical Domain	Theme	Facilitator	Barrier	Illustrative quotes
Knowledge	Clear understanding of medication error definitions	✓		*“Medication errors include issues like incorrect dosing frequency or prescribing contraindicated medications.”(Physician 3)*
	Clear distinction between medication errors and adverse drug reactions	✓		*“Adverse reactions refer to situations where, under normal dosage and administration of a medication, an effect unrelated to the therapeutic purpose occurs. Medication errors, however, explicitly involve the presence of mistakes, with human error being a primary contributing factor.”(Pharmacist 9)*
	Limited awareness of medication error reporting systems/policies		✓	*“Our hospital lacks a dedicated medication error reporting system—only adverse event protocols exist. I’m uncertain about the specific reporting process.”(Physician 7)*
	Unfamiliarity with expert consensus		✓	*“I’m not aware of any expert consensus on this topic.”(Physician 1)*
Skills	Competence to identify and report medication errors	✓	✓	*“Identifying errors requires systematic checks, such as PDA scanning.”(Nurse 6)* *“I’ve never reported a medication error.”(Physician 2)*
	Insufficient training experiences		✓	*“I haven’t received any training on medication errors. We usually rely on experience or advice from colleagues.”(Physician 6)*
	Demand for skill-building training on error reporting	✓		*“Training would be very helpful, particularly for nurses administering medications*.*”(Nurse 1)*
Social/Professional Role & Identity	Professional obligation	✓		*“It’s our duty to ensure patient safety, and reporting errors helps prevent harm to patients.”(Nurse 4)*
Beliefs in Capabilities	Confidence in reporting capability	✓	✓	*“I’m confident in my ability to report errors, thanks to years of experience.”(Pharmacist 4)* *“I genuinely lack experience in reporting errors and am not entirely clear on how the reporting system functions in practice, which undermines confidence.”(Physician 4)*
	Perceived difficulties in reporting		✓	*“Heavy workloads leave little time for reporting.”(Pharmacist 5)*
	Resolution of reporting difficulties		✓	*“Establishing a specialized role dedicated exclusively to error reporting.”(Pharmacist 5)*
Optimism	Optimism about reporting	✓	✓	*“About 20-30%. This is because medication errors can stem from a multitude of factors.”(Pharmacist 5)* *“I’d say about 80-90% certainty that there’s no issue. After all, no one can guarantee 100% perfection.”(Nurse 2)*
Beliefs about Consequences	Belief that reporting improves patient safety	✓		*“Reporting errors helps reduce mistakes and improves patient safety.”(Pharmacist 3)*
	Lack of feedback after reporting		✓	*“I have reported medication errors several times before, but after submitting them, there was no follow-up. This made me doubt the effectiveness of the entire system. Over time, it made me reluctant to waste time reporting.”(Pharmacist 7)*
	Fear of damaging workplace relationships		✓	*“If I report someone else’s error, they might feel targeted. If they find out, it would definitely affect our working relationship.”(Nurse 4)*
	Concerns about negative impacts on career progression		✓	*“Once a serious medication error occurs, reporting it might lead to a loss of trust from colleagues and patients, or even jeopardize my position and reputation at the hospital. The hospital might also investigate my work, which could impact my professional title promotion and career advancement.”(Physician 9)*
Reinforcement	Incentives for reporting	✓		*“Reporting errors should be counted as part of our workload credits and linked to professional evaluations.”(Pharmacist 1)*
Intentions	Intention to report errors	✓	✓	*“I would definitely report any medication error, as it’s our responsibility.”(Nurse 4)* *“I used to report errors, but now I only report serious ones.”(Pharmacist 7)*
Goals	Commitment to enhancing patient safety	✓		*“Reporting mitigates risks and safeguards patients*.*”(Physician 8)*
	Improving medication management and policies	✓		*“Reporting errors helps us identify and fix gaps in our medication processes.”(Pharmacist 7)*
	Prioritization of other tasks over error reporting		✓	*“To be honest, reporting medication errors is something I leave until the very end of all my tasks. I only find time to do it after finishing all my clinical work, right before clocking out. Like, ‘Oh, I still have a medication error to report today.’ It’s not like I’d report it immediately when an error occurs.”(Pharmacist 9)*
Memory, Attention, and Decision Processes	Selective reporting based on error severity		✓	*“Reporting depends on how serious the mistake is. For critical errors that could harm a patient, we report immediately. But for minor issues—like a near-miss caught before dispensing—there’s less urgency. Sometimes those get delayed or even overlooked if workloads are heavy.”(Pharmacist 5)*
	Forgetting to report		✓	*“Sometimes, when the counter is extremely busy, I forget about reporting after stepping away. The biggest challenge now is simply forgetting.”(Pharmacist 7)*
Environmental Context and Resources	Inadequate reporting systems		✓	*“The reporting process is too manual and time-consuming.”(Pharmacist 3)*
	Organizational culture	✓	✓	*“No, at most there might be some worry—it’s human nature. If you make a mistake, it’s natural to feel concerned. You wonder: Will this affect the department, the team, or my own work? But if everyone steps in quickly to address it, those worries fade fast.”(Nurse 2)*
Social Influences	Leadership attitudes		✓	*“Our department leader focuses on resolving issues rather than assigning blame or punishment. That’s why I feel safe reporting errors—this approach is crucial.”(Nurse 4)*
	Concerns about patient reactions		✓	*“Patient reactions matter. Given today’s strained physician-patient relationships, disclosing human errors that create safety risks is daunting. Medication errors are categorized into nine severity levels. If a patient suffers harm, they might escalate complaints. This fear of confrontation weighs heavily on us psychologically.”(Pharmacist 5)*
Emotion	Emotional influences		✓	*“My mood affects my willingness to report errors.”(Pharmacist 5)*
	Stress		✓	*“Personally, stress doesn’t sway me. I firmly prioritize work responsibilities during shifts—home matters can wait until after work. But everyone’s different.”(Physician 1)*
Behavioral Regulation	Behavioral plans	✓		*“First, it’s essential to strengthen medication-related expertise and strictly adhere to verification protocols to optimize medication processes. For any uncertainties, it’s important to consult physicians or seek guidance from senior nurses.”(Nurse 5)*
	Improvement suggestions	✓		*“It’s essential to train clinical staff and raise their awareness of reporting-this is likely the most important step.”(Physician 3)*

### 3.1 Knowledge

#### 3.1.1 Clear understanding of medication error definitions

Most participants demonstrated a clear understanding of medication errors and provided illustrative examples.

“Medication errors include issues like incorrect dosing frequency or prescribing contraindicated medications. For example, I mistakenly prescribed dapagliflozin three times daily instead of once daily for a diabetic patient. This error remained undetected until discharge.”(Physician 3)

#### 3.1.2 Clear distinction between medication errors and adverse drug reactions

All interviewees were able to accurately distinguish between medication errors and adverse drug reactions.

“Adverse reactions refer to situations where, under normal dosage and administration of a medication, an effect unrelated to the therapeutic purpose occurs. Medication errors, however, explicitly involve the presence of mistakes, with human error being a primary contributing factor.”(Pharmacist 9)

#### 3.1.3 Limited awareness of reporting systems/policies

Physicians exhibited limited familiarity with institutional reporting protocols, while nurses and pharmacists demonstrated greater awareness.

“Our hospital lacks a dedicated medication error reporting system—only adverse event protocols exist. I’m uncertain about the specific reporting process.”(Physician 7)

“We should contact the head nurse. The head nurse is aware of such matters and will provide you with a form. After completing the form, detailing the incident and the corresponding patient information, submit it back to the head nurse. The head nurse will then report it to the Nursing Department.”(Nurse 4)

#### 3.1.4 Unfamiliarity with expert consensus

Physicians and nurses generally lacked knowledge about expert consensus on medication error reporting, while some pharmacists were more informed.

“I’m not aware of any expert consensus on this topic.”(Physician 1)

“To report, since I’ve received systematic training, I know there is a expert consensus for medication errors and a specific form. I also know where to download that form.”(Pharmacist 5)

### 3.2 Skills

#### 3.2.1 Competence to identify and report medication errors

Most healthcare professionals believed they had the ability to identify medication errors, but the majority, especially physicians, had never actively reported such errors.

“Identifying errors requires systematic checks, such as PDA scanning.”(Nurse 6)

“I’ve never reported a medication error.”(Physician 2)

#### 3.2.2 Insufficient training experiences

Systematic training on error reporting was notably absent.

“I have not received any training on medication errors. We usually rely on experience or advice from colleagues.”(Physician 6)

#### 3.2.3 Demand for skill-building training on error reporting

Participants emphasized needs for updated clinical knowledge, communication skills, and safety awareness training.

“Training would be very helpful, particularly for nurses administering medications.”(Nurse 1)

“I believe the most urgent need is to improve safety awareness … Regular training on medication safety, such as identifying errors, recognizing common risks, and learning prevention strategies, would help us better understand our responsibilities. Additionally, training on how to promptly report and correct errors is equally vital.”(Physician 7)

### 3.3 Social/professional role and identity

#### 3.3.1 Professional obligation

Interviewees believed that reporting medication errors was an important part of ensuring patient safety.

“As pharmacists, our primary responsibility is to ensure patient safety, and reporting medication errors is a key part of that.”(Pharmacist 5)

“It’s our duty to ensure patient safety, and reporting errors helps prevent harm to patients.”(Nurse 4)

### 3.4 Beliefs in capabilities

#### 3.4.1 Confidence in reporting capability

Nurses and pharmacists generally felt confident in their ability to report medication errors, while physicians lacked confidence due to limited reporting experience.

“I’m confident in my ability to report errors, thanks to years of experience.”(Pharmacist 4)

“I genuinely lack experience in reporting errors and am not entirely clear on how the reporting system functions in practice, which undermines confidence.”(Physician 4)

“I believe I am fully capable of reporting medication errors. Through years of clinical practice, I’ve developed a habit of quick response. Once a medication error is identified, I take immediate action to ensure patient safety.”(Nurse 8)

#### 3.4.2 Perceived difficulties in reporting

Some healthcare staff encountered challenges in reporting medication errors due to workload pressures, communication barriers, staffing deficits, and time constraints, while certain nurses reported minimal difficulties in this process.

“Heavy workloads leave little time for reporting.”(Pharmacist 5)

“I still feel there’s a gap in theoretical knowledge in this area.…I think communication skills are indeed a significant area that needs improvement.”(Pharmacist 3)

“There’s no real difficulty. I think since the hospital has established these protocols, if a mistake happens, you just follow the standard procedure.”(Nurse 2)

#### 3.4.3 Resolution of reporting difficulties

Interviewees suggested that dedicated positions, leadership support, and non-punitive environments could help overcome reporting difficulties.

“Establishing a specialized role dedicated exclusively to error reporting.”(Pharmacist 5)

“I think the most critical factor is leadership commitment. If the leaders and supervisors take this seriously, then the work can move forward.”(Physician 3)

“Establishing incentive mechanisms or reducing workload burdens could encourage healthcare professionals to actively report errors… Additionally, hospitals should foster a non-punitive environment where staff feel safe to report errors, knowing they can learn from mistakes without fear of punishment.”(Nurse 7)

### 3.5 Optimism

#### 3.5.1 Optimism about reporting

Nurses generally demonstrate a higher level of optimism and are more likely to successfully report errors. Pharmacists show moderate optimism; while physicians tend to be less optimistic.

“Honestly, I’m not very optimistic. Our department operates at a fast pace with high pressure, and we’re often too busy to pay attention. Even when we notice a medication error, there’s rarely time to document and report it in detail.”(Physician 5)

“About 20%–30%. This is because medication errors can stem from a multitude of factors.”(Pharmacist 5)

“To be honest, I’m not optimistic. Our department operates at a fast pace with high pressure, often leaving no time to address such issues.”(Physician 5)

### 3.6 Beliefs about consequences

#### 3.6.1 Belief that reporting improves patient safety

Reporting medication errors enhances healthcare professionals’ vigilance, enabling them to avoid similar mistakes in the future. It also facilitates the timely resolution of issues, thereby improving patient safety.

“Reporting errors helps reduce mistakes and improves patient safety.”(Pharmacist 3)

“Reporting errors alerts everyone to potential issues and helps prevent similar mistakes.”(Nurse 3)

#### 3.6.2 Lack of feedback after reporting

The interviewed healthcare professionals, particularly pharmacists, pointed out that the lack of feedback is the main reason for the decline in pharmacists’ motivation to report.

“I have reported medication errors several times before, but after submitting them, there was no follow-up. This made me doubt the effectiveness of the entire system. Over time, it made me reluctant to waste time reporting.”(Pharmacist 7)

#### 3.6.3 Fear of damaging workplace relationships

Reporting errors could strain relationships with colleagues, especially if the error involved someone else.

“If I report someone else’s error, they might feel targeted. If they find out, it would definitely affect our working relationship.”(Nurse 4)

#### 3.6.4 Concerns about negative impacts on career progression

Some participants feared that reporting medication errors could jeopardize their career progression, reputations, and promotion opportunities, potentially triggering investigations that threaten their professional standing.

“Once a serious medication error occurs, reporting it might lead to a loss of trust from colleagues and patients, or even jeopardize my position and reputation at the hospital. The hospital might also investigate my work, which could impact my professional title promotion and career advancement.”(Physician 9)

### 3.7 Reinforcement

#### 3.7.1 Incentives for reporting

Some interviewees suggested that reporting errors should be linked to workload credits, professional evaluation, and financial rewards.

*“Reporting errors should be counted as part of our workload* credits *and linked to professional evaluations.”(Pharmacist 1)*

“We need incentives similar to those for reporting adverse events.”(Physician 1)

### 3.8 Intentions

#### 3.8.1 Intention to report errors

Nurses generally perceived reporting as a professional duty and demonstrated a strong propensity for proactive reporting, while physicians showed limited reporting willingness. Conversely, pharmacists tended to prioritize reporting based on error severity levels.

“I would definitely report any medication error, as it’s our responsibility.”(Nurse 4)

“I used to report errors, but now I only report serious ones.”(Pharmacist 7)

“I think, It’s not about rewards or punishments affecting my decision to report; it’s more that my awareness of reporting medication errors is not very strong. I did not immediately realize I should report a medication error.”(Physician 2)

### 3.9 Goals

#### 3.9.1 Commitment to enhancing patient safety

Healthcare professionals generally agreed that the goal of reporting medication errors is to enhance patient safety and prevent serious consequences.

“Reporting mitigates risks and safeguards patients.”(Physician 8)

#### 3.9.2 Improving medication management and Policies

Respondents noted that medication error reporting facilitates process improvements, enhances medication safety and management efficiency, and informs evidence-based policy development.

“Reporting errors helps us identify and fix gaps in our medication processes.”(Pharmacist 7)

“Analyzing error reports helps us identify high-risk drugs and improve management policies.”(Pharmacist 8)

#### 3.9.3 Prioritization of other tasks over error reporting

Some participants reported that medication error reporting is frequently deprioritized in favor of immediate clinical responsibilities, often addressed only after primary tasks are completed.

“To be honest, reporting medication errors is something I leave until the very end of all my tasks. I only find time to do it after finishing all my clinical work, right before clocking out. Like, Oh, ‘I still have a medication error to report today’. It’s not like I’d report it immediately when an error occurs.”(Pharmacist 9)

### 3.10 Memory, attention, and decision processes

#### 3.10.1 Selective reporting based on error severity

Some interviewed pharmacists pointed out that the timing of reporting medication errors is often linked to the severity of the error.

“Reporting depends on how serious the mistake is. For critical errors that could harm a patient, we report immediately. But for minor issues—like a near-miss caught before dispensing—there’s less urgency. Sometimes those get delayed or even overlooked if workloads are heavy.”(Pharmacist 5)

#### 3.10.2 Forgetting to report

Interviewed pharmacists acknowledged the significance of error reporting but noted high workload pressures and time-sensitive duties often resulted in underreporting. Conversely, nursing staff highlighted that collaborative team environments fostered proactive error disclosure through mutual support mechanisms.

“Sometimes, when the counter is extremely busy, I forget about reporting after stepping away. The biggest challenge now is simply forgetting.”(Pharmacist 7)

“I think it’s impossible for me [to forget]. Once you’ve made a mistake, you’d naturally feel fearful—how could you not report it? … If an error happened to me, no matter what, I’d report it immediately. After all, there’s a team here to support you.”(Nurse 2)

### 3.11 Environmental context and resources

#### 3.11.1 Inadequate reporting systems

Some interviewed pharmacists pointed out that the medication error reporting process is overly complex and cumbersome, lacking a user-friendly system. Physicians mentioned additional barriers, such as the reporting system’s limited accessibility.

“The reporting process is too manual and time-consuming.”(Pharmacist 3)

“The reporting system is not available on all computers; it’s only accessible on specific machines connected to an external network. You have to wait until that dedicated computer is free to file a report.”(Physician 1)

#### 3.11.2 Organizational culture

Interviewees acknowledged psychological pressure when reporting medication errors. However, anxiety lessens with swift team intervention and resolution.

“No, at most there might be some worry—it’s human nature. If you make a mistake, it’s natural to feel concerned. You wonder: Will this affect the department, the team, or my own work? But if everyone steps in quickly to address it, those worries fade fast.”(Nurse 2)

### 3.12 Social influences

#### 3.12.1 Leadership attitudes

Interviewees emphasized the significant influence of department leadership attitudes on medication error reporting. When leaders prioritize problem-solving over punishment, staff are more willing to report errors.

“Our department leader focuses on resolving issues rather than assigning blame or punishment. That’s why I feel safe reporting errors—this approach is crucial.”(Nurse 4)

“Leadership recognition is a major driver. For instance, even if reporting earned a trivial monetary reward, most would not care about the money itself. What matters is that it validates your contribution to the team and shows leadership acknowledges your effort. To me, that recognition is vital.”(Pharmacist 7)

#### 3.12.2 Concerns about patient reactions

Some participants indicated that for minor medication errors (e.g., non-harmful substitutions), healthcare professionals often withhold disclosure and skip reporting, deeming it unnecessary. However, in severe cases threatening patient safety, reporting is treated as mandatory, despite concerns about exacerbating doctor-patient tensions.

“Patient reactions matter. Given today’s strained physician-patient relationships, disclosing human errors that create safety risks is daunting. Medication errors are categorized into nine severity levels. If a patient suffers harm, they might escalate complaints. This fear of confrontation weighs heavily on us psychologically.”(Pharmacist 5)

### 3.13 Emotion

#### 3.13.1 Emotions influences

Interviewees highlighted that competing priorities and emotional states can undermine focus and proactive engagement in medication error reporting.

“My mood affects my willingness to report errors.”(Pharmacist 5)

#### 3.13.2 Stress

The impact of stress on medication error reporting varies among individuals. While some medical professionals acknowledged that workload pressures significantly hinder their reporting behaviors, others maintained their ability to prioritize error reporting despite stress.

“Yes, stress affects reporting. For example, when patients or families are pressuring you to complete tasks, or when you’re multitasking during medication preparation, errors like misreading prescriptions can easily occur. Feeling overwhelmed, you might delay reporting or even deprioritize it to address more urgent issues.”(Nurse 3)

“Personally, stress does not sway me. I firmly prioritize work responsibilities during shifts—home matters can wait until after work. But everyone’s different.”(Physician 1)

### 3.14 Behavioral regulation

#### 3.14.1 Behavioral plans

Nurses emphasized strengthening individual expertise and consulting colleagues to resolve prescription uncertainties, while pharmacists and physicians highlighted the need to shift mindsets toward proactive reporting.

“First, it’s essential to strengthen medication-related expertise and strictly adhere to verification protocols to optimize medication processes. For any uncertainties, it’s important to consult physicians or seek guidance from senior nurses.”(Nurse 5)

“First, we need clear definitions and scope of medication errors—this must be standardized. Through initiatives like this interview, we should heighten awareness, implement rigorous checks, and minimize oversight risks.”(Physician 2)

#### 3.14.2 Improvement suggestions

Interviewees emphasized that improving medication error reporting systems requires fundamental shifts in workplace culture and practices, including better training, leadership support, and simplified reporting processes.

“Previous attempts at reform were difficult because the habit of reporting was never fundamentally cultivated. I suggest embedding these principles into new employee onboarding training. Leadership must prioritize this in departmental performance metrics and staff recognition, and institutional frameworks need restructuring.”(Pharmacist 5)

“It’s essential to train clinical staff and raise their awareness of reporting-this is likely the most important step.”(Physician 3)

“The first issue lies at the hospital and management level. You must establish a medication error monitoring and reporting system. Start with clear policies, detailed plans, and definitions. Create a simple, user-friendly reporting process and define how to handle reports afterward. Second, improve medication management systems. Strengthen training for medical professionals. Enhance patient communication. Most crucially, adopt information technology-replace paper forms with computerized systems, similar to adverse event platforms, and conduct regular reviews for process refinement.”(Physician 1)

## 4 Discussion

This study elucidates the multifaceted determinants of medication error reporting among healthcare professionals in China using the Theoretical Domains Framework (TDF). Through rigorous analysis, we identified 21 barriers and 15 facilitators to reporting. To our knowledge, this is the first in-depth investigation applying the TDF to systematically analyze medication error behaviors within Chinese healthcare workforce. The findings underscore a dynamic interplay of individual, organizational, and cultural factors that collectively influence reporting behaviors, revealing systemic gaps and actionable pathways for improvement.

Participants demonstrated robust conceptual knowledge of medication errors and their distinction from adverse drug reactions. However, physicians exhibited markedly lower awareness of institutional reporting protocols than nurses and pharmacists. This role-specific policy knowledge gap aligns with studies in hierarchical healthcare systems, such as those in Qatar and the UAE, where profession-segregated workflows similarly hinder standardized reporting practices (Alqubaisi et al., 2016; Stewart et al., 2018). Compounding these knowledge deficits, systemic shortcomings in training-especially in communication strategies and high-risk medication management-were identified as key skill-related barriers. While evidence confirms targeted training enhances reporting rates (Force et al., 2006; Lederman et al., 2013), the persistent misalignment between China’s regulatory frameworks (e.g., Quality Control Indicators for Pharmaceutical Management) and frontline implementation underscores an urgent need for policy operationalization. This disconnection perpetuates preventable risks by fostering inconsistencies between mandated standards and clinical execution.

The TDF domain social/professional role and identity revealed cultural tensions inherent to error reporting. In our study, participants universally acknowledged reporting as a professional obligation; however, we found that cultural norms emphasizing workplace harmony and hierarchical deference introduced unique barriers. These dynamics were especially prominent in contexts influenced by Confucian values, aligning with broader observations (Badanta et al., 2022). Fear of damaging colleague relationships and career repercussions deterred transparency, mirroring findings in other East Asian contexts where collectivist values conflict with error disclosure (Loewenbrück et al., 2016). Although most professionals expressed confidence in their ability to identify and report errors, practical obstacles—including excessive workloads, communication breakdowns, staffing shortages, and time constraints—significantly impeded reporting. We identified systemic issues exacerbating these challenges—notably inadequate feedback mechanisms, fear of punitive repercussions, and insufficient incentives diminishing motivation. This pattern in our data is corroborated by existing literature on blame culture and reward deficiencies (Hamed and Konstantinidis, 2022). These findings collectively underscore the tension between individual responsibility and organizational-cultural constraints, necessitating interventions that address both behavioral and systemic determinants of reporting efficacy.

Qualitative findings highlight a strong consensus among healthcare professionals for systematically linking medication error reporting behaviors to performance metrics, career advancement pathways, and financial incentives. This demand aligns with international evidence demonstrating that blended economic-educational interventions—such as monetary rewards paired with competency training—significantly elevate adverse event reporting rates (from 1.6% to 9.0%) and near-miss reporting (from 6% to 27%) (Scott et al., 2011),. These outcomes confirm the critical need for dual reinforcement mechanisms that bridge economic incentives and professional development imperatives validating practitioners’ demands for dual economic-professional reinforcement. Leadership practices significantly moderated these dynamics: non-punitive leadership fostered psychological safety, whereas absent feedback mechanisms eroded trust. These observations extend the TDF’s applicability to collectivist systems, where Social Influences and Beliefs About Consequences dominantly shape decision-making. Structural inefficiencies, such as cumbersome reporting systems and competing clinical priorities, further impeded timely reporting. The prevalent prioritization of severe errors over near-misses-contrary to comprehensive reporting policies-and pharmacists’ demotivation due to post-reporting inertia reflect systemic flaws observed cross-culturally (Alqubaisi et al., 2016).

Profession-specific variations in reporting barriers were pronounced. Nurses exhibited higher proactive reporting rates within team-driven environments, consistent with nursing culture’s emphasis on collective accountability (Wilson, 2013; Kristensen et al., 2015). Conversely, pharmacists, despite superior pharmacological expertise, faced reporting delays due to workload saturation, while physicians’ low confidence stemmed from time constraints and unfamiliarity with reporting systems. Although physicians generally acknowledged their capacity to identify medication errors, urgent clinical priorities and unfamiliarity with reporting mechanisms frequently obstructed timely disclosures (Takhtinejad et al., 2024). These findings partially diverge from prior studies (Alqubaisi et al., 2016; Stewart et al., 2018), highlighting how China’s unique clinical pressures—such as high patient volumes and hierarchical workflows—exacerbate profession-specific challenges.

While this study provides critical insights into medication error reporting behaviors, several limitations warrant consideration. First, the purposive sampling focused on professionals from Shanxi Province, which may limit generalizability to other regions with distinct healthcare infrastructures or cultural dynamics. Second, self-reporting bias could affect data reliability, as participants might underreport sensitive issues (e.g., fear of career repercussions) despite confidentiality assurances-a concern corroborated by physicians’ expressed reluctance to disclose errors involving colleagues. Third, while thematic saturation was rigorously pursued, the sample size (n = 27) and profession-specific recruitment (9 nurses, 10 pharmacists, 8 physicians) may inadequately represent the full spectrum of hierarchical interactions in China’s diverse hospital tiers. Finally, the TDF’s theoretical framing, though comprehensive, risks overlooking contextual factors unique to China’s collectivist clinical environments, such as guanxi (interpersonal networks), which were implicitly reported but not systematically analyzed. These constraints highlight the need for mixed-methods approaches in future research to triangulate findings across broader geographical and institutional contexts.

## 5 Conclusion

In conclusion, this study advances the TDF in non-western contexts, revealing how China’s institutional and cultural landscapes shape medication error reporting. By integrating knowledge reinforcement, systemic optimization, and cultural transformation, healthcare institutions can evolve reporting from a fragmented obligation into a sustainable driver of patient safety. The path forward demands cross-professional collaboration, policy enforcement, and a commitment to fostering environments where transparency is both safe and rewarded.

## Data Availability

The raw data supporting the conclusions of this article will be made available by the authors, without undue reservation.
