# Supplementary material for: Exploring multidimensional determinants of medication error reporting in China: a qualitative study using the theoretical domains framework
Source: Front Pharmacol. 2025 Jun 10;16:1590794. doi: 10.3389/fphar.2025.1590794 (PMC12185516; doi:10.3389/fphar.2025.1590794)
Supplement: Supplementary file 1 [file Table1.docx]

Supplementary Material

Table S1 TDF-based interview guide for healthcare professionals' medication error reporting behavior

| Theoretical Domain | Definition​​ ​​ | Specific Questions​​ | Prompts |
| --- | --- | --- | --- |
| **Knowledge​**​ | Healthcare professionals' understanding of medication error reporting, including definitions, reporting procedures, and reference guidelines. | ① Can you describe what constitutes a medication error? ② Are you aware of how to identify and report medication errors in clinical practice? ③ What guidelines/consensus statements/institutional protocols do you follow when reporting medication errors? | What distinguishes a medication error from an adverse drug reaction? |
| **Skills​**​ | Competencies required for medication error reporting, including specific skills and training needs. | ① Do you find it easy or challenging to report medication errors? ② What specific skills do you need to report medication errors in your role as a physician/pharmacist/nurse? ③ Can you suggest any skill-specific training that could improve your medication error reporting? | • What makes reporting medication errors easier or harder?  • What are your thoughts on training? Do you feel it is necessary?  *Training topics: Identifying errors, managing errors, communication with patients/staff, decision-making.* |
| **Social/Professional Role** & **Identity​**​ | Perception of one's professional obligations in medication error reporting. | ① Do you consider medication error reporting to be part of your professional responsibility? | • Could you explain further? |
| Beliefs in Capabilities​ | Confidence in one's ability to successfully report medication errors. | ① How confident are you in your ability to report medication errors? ② What difficulties do you encounter when reporting? Who could help address these challenges? | • What drives your motivation (internal/external)?  • Could you give an example?  *Triggers: Self-reminders, colleague reminders.* |
| **Optimism​**​ | Belief that reporting will lead to positive outcomes. | ① How likely do you think you are to successfully report a medication error? | • Could you explain your reasoning? |
| **Beliefs about Consequences​**​ | Perceived outcomes and impacts of reporting/not reporting errors. | ① What do you think would happen if you report a medication error? ② What might happen if you don't report it? ③ What benefits/costs do you associate with medication error reporting? | • Impacts on yourself, patients, colleagues, or the organization (short- and long-term). |
| **Reinforcement​**​ | Incentives or disincentives influencing reporting behaviors. | ① What conditions might encourage or discourage you to report medication errors? | • Incentives, recognition, or other motivators. |
| **Intentions​**​ | Willingness to engage in error reporting. | ① Do you intend to continue reporting medication errors? | • If yes, what challenges do you foresee? If no, why? |
| **Goals​**​ | Prioritization of error reporting relative to other objectives. | ① What do you perceive as the primary goal of medication error reporting? ② Are there competing priorities that might prevent you from reporting? | • If yes, what challenges do you foresee? If no, why? |
| **Memory, Attention, and Decision Processes​**​ | Cognitive factors affecting reporting. | ① What typically triggers your decision to report an error? ② Under what circumstances might you forget to report an error? | • What do you think drives these patterns? |
| **Environmental Context and Resources​**​ | Institutional/environmental facilitators or barriers. | ① How does your work environment influence your reporting behaviors? ② What environmental factors (e.g., colleagues, leadership) hinder or facilitate reporting? | • Physical vs. cultural factors: Staff shortages, lack of reporting systems, feedback, or incentives. |
| **Social Influences​**​ | Interpersonal/organizational pressures impacting reporting. | ① Who influences your decision to report (e.g., colleagues, supervisors)? How? ② How do patient/family/public expectations affect your reporting decisions? | • In what contexts and to what extent? |
| **Emotion​**​ | Psychological states during reporting. | ① How do emotions influence your willingness to report? ② How does work stress impact your reporting behaviors? | • Could you describe a specific example? |
| **Behavioral Regulation​**​ | Strategies to manage reporting behaviors. | ① What plans do you make to ensure error reporting? ② If you wanted to improve your reporting practices, how would you approach this? | • What actions or conditions would enable these changes? |
